# Smart Biosensor for Breast Cancer Survival Prediction Based on Multi-View Multi-Way Graph Learning

**DOI:** 10.3390/s24113289

**Published:** 2024-05-21

**Authors:** Wenming Ma, Mingqi Li, Zihao Chu, Hao Chen

**Affiliations:** School of Computer and Control Engineering, Yantai University, Yantai 264005, China; lmq135831@s.ytu.edu.cn (M.L.); czh1126940012@s.ytu.edu.cn (Z.C.); 202200358067@s.ytu.edu.cn (H.C.)

**Keywords:** biosensors similarity graph, gene interaction graph, graph learning, machine learning, smart biosensor

## Abstract

Biosensors play a crucial role in detecting cancer signals by orchestrating a series of intricate biological and physical transduction processes. Among various cancers, breast cancer stands out due to its genetic underpinnings, which trigger uncontrolled cell proliferation, predominantly impacting women, and resulting in significant mortality rates. The utilization of biosensors in predicting survival time becomes paramount in formulating an optimal treatment strategy. However, conventional biosensors employing traditional machine learning methods encounter challenges in preprocessing features for the learning task. Despite the potential of deep learning techniques to automatically extract useful features, they often struggle to effectively leverage the intricate relationships between features and instances. To address this challenge, our study proposes a novel smart biosensor architecture that integrates a multi-view multi-way graph learning (MVMWGL) approach for predicting breast cancer survival time. This innovative approach enables the assimilation of insights from gene interactions and biosensor similarities. By leveraging real-world data, we conducted comprehensive evaluations, and our experimental results unequivocally demonstrate the superiority of the MVMWGL approach over existing methods.

## 1. Introduction

Biosensors play a pivotal role across diverse medical research domains and applications, facilitating the detection of specific biological reactions or occurrences [1]. In the realm of cancer detection, they serve as indispensable tools for identifying biomarkers associated with cancerous cells or tumors, such as aberrant levels of protein or gene expression within targeted cells [2]. Over recent years, breast cancer has emerged as a significant health concern for women globally, characterized by a relatively high mortality rate ranging from 10 to 15 per 100,000 in most nations [3]. This disease stems from genetic abnormalities that lead to the production of faulty proteins, culminating in uncontrolled cell proliferation and the formation of breast tumors [4]. Breast cancer is categorized into various stages based on factors such as tumor size, location, and other parameters, each necessitating tailored treatment modalities [5]. Accurately predicting survival time is imperative for guiding clinicians in prognosis and devising personalized treatment strategies. However, even seasoned experts may encounter challenges in this endeavor. The conventional approach involves integrating machine learning models into smart biosensors, wherein treatment and sensor data from cancer patients are collected to design models that discern survival patterns [6].

Smart biosensors leverage machine learning techniques to establish correlations between survival time, sensor data, and biological attributes like age, race, weight, tumor size, and gene expression levels [7]. Nonetheless, traditional machine learning algorithms [8,9] necessitate extensive preprocessing of features, including selection, combination, transformation, and handling missing data [10], often resulting in suboptimal generalization ability and practicality, thereby impeding accurate outcomes.

In recent years, deep learning methodologies have made significant strides across various domains [11,12,13], offering promise when integrated into smart biosensors for cancer prediction. Deep learning offers several advantages, including the automatic extraction of valuable representations from raw data through tailored network architectures for specific tasks [14]. For example, CNNs (Convolutional Neural Networks) are adept at learning features from grid data like images [15,16], while LSTMs (Long Short-Term Memory networks) excel at extracting features from sequential data [17,18]. Another notable advantage of deep learning is its innate capability to amalgamate multi-modal features into a cohesive representation, thereby harnessing all pertinent information from diverse data types [19].

Biosensors serve a crucial function in measuring gene expression levels, which are essential for predicting survival time in breast cancer [20]. However, training machine learning algorithms require thousands of gene expression levels. Yet, the etiology of breast cancer is complex, involving interactions between genes rather than mere simultaneous damage to select genes. Regrettably, deep learning techniques are ill-suited for handling graph data, such as gene interactions, thus precluding the utilization of this vital information [21,22]. Moreover, most breast cancer datasets are relatively small, and the assumption in deep learning that data samples are independent and identically distributed (IID) often leads to overfitting. By considering patient correlations, more valuable information can be gleaned even with limited data. However, these correlations are represented as a graph structure, which conventional deep learning algorithms cannot accommodate.

In recent years, Graph Neural Networks (GNNs) have garnered substantial attention for learning tasks involving graph data structures. GNNs iteratively update node representations by aggregating information from neighboring nodes, efficiently leveraging both structural and feature information. Various GNN architectures [23,24,25,26] exhibit distinct characteristics and are employed in diverse scenarios. Some recent studies advocate utilizing a gene interaction graph [27], with gene expression serving as input, to predict breast cancer survival [28]. However, these methods predominantly rely on supervised and graph-level learning, overlooking the potential of semi-supervised learning in exploiting patient relationships, particularly in datasets with limited samples.

Nevertheless, existing smart biosensors grapple with inherent limitations that warrant attention. Primarily, they often exhibit limited computing power and memory, rendering it challenging to execute deep learning or graph neural models comprising thousands or millions of parameters. Additionally, even if certain biosensors can support deep learning models, they fail to capitalize on patient connections, as a solitary biosensor can only measure the gene expression of one individual at a time and cannot execute a graph neural model treating multiple patients as a coherent graph structure. Lastly, extant graph-based methods predominantly harness relationships between genes, neglecting the opportunity to derive more potent representations from both gene and patient graphs.

To surmount these challenges, we propose a novel smart biosensor architecture employing Multi-view Multi-way Graph Learning (MVMWGL) to predict the survival time of breast cancer patients. Comprising three core components—the *Reprentator*, *Learning center*, and *Predictor*, all rooted in a traditional architecture—the biosensor system operates as follows: initially, gene expression levels are collated from the biosensor’s processor and transmuted into raw patient features in vector format. These raw features are then transmitted to the learning center, which aggregates gene features from numerous patients to execute graph neural networks and periodically trains the models. Subsequently, the biosensor can instantaneously obtain a high-level representation from the learning center and periodically download the updated prediction model. Leveraging the predictor and high-level representation, the biosensor can accurately prognosticate patient survival. The hallmark of our proposed smart biosensor lies in its innovative Learning Center, which deploys MVMWGL models from both gene interaction and patient similarity perspectives, employing three distinct learning paradigms to yield more precise predictions, particularly in datasets with limited samples.

The main contributions of this study are summarized as follows:

- We introduce a novel biosensor architecture that incorporates innovative intelligent components enabling the deployment of complex machine learning models in both local and remote settings. These intelligent components comprise the *Representator*, *Learning Center*, and *Predictor*.
- Within the *Learning Center*, we employ graph neural networks to extract information from gene interaction and biosensor similarity graphs. Utilizing multi-way learning, we enhance the model’s capacity to capture diverse representations from small datasets.
- To showcase the superiority of our proposed learning method in the *Learning Center*, we compare its performance against traditional machine learning, deep learning, and GNN-based methods using real-world breast cancer data.
- Additionally, we conduct exploratory experiments to analyze key characteristics of our proposed learning method, providing insights into its underlying mechanisms and potential applications.

## 2. Related Work

In this section, we present a thorough overview of relevant studies focusing on smart biosensors and methods for predicting cancer survival. We begin by summarizing the technologies that merge biosensors with machine learning methodologies. Following that, we explore three main categories of machine learning approaches for cancer prediction: traditional methods, deep learning techniques, and graph neural network models. Finally, we demonstrate the advantages of our proposed method through comparison with other conventional approaches.

### 2.1. Machine Learning Enabled Smart Biosensors

In recent years, there has been a growing trend of utilizing machine learning models in conjunction with biosensors to improve detection and prediction capabilities. Kadian et al. offer an extensive review of machine learning technologies applied to enhance the intelligent features of noninvasive biosensors [29]. They outline various machine learning algorithms employed in smart biosensors for clinical decision-making, encompassing both traditional and deep learning methods. Additionally, they highlight common applications of machine learning in non-invasive biosensors, such as portable and wearable devices, while addressing the associated research challenges.

Ballard et al. present a comprehensive overview of advanced computational sensing systems that integrate computation and machine learning techniques [30]. They delineate the design workflow of smart biosensors into several stages, including data acquisition, model input–output determination, cost function specification, and hardware redesign.

Zhang et al. delve into the augmentation of noninvasive biosensors through machine learning algorithms across various domains, including disease diagnosis, health monitoring, and food safety [31]. They provide insights into biomedical signal processing and the utilization of both non-neural and neural network algorithms in biosensors, along with an analysis of the strengths and weaknesses of different algorithms.

Jin et al. review recent advancements in smart wearable biosensors incorporating machine learning methods [32]. They advocate for the development of flexible electronic materials to seamlessly integrate machine learning models and stress the importance of sensors capable of adaptive learning through convenient human–machine interaction.

Cui et al. examined the recent application of advanced machine learning algorithms in biosensors [33]. They argue that machine learning facilitates chemometrics, enhancing biosensor intelligence, and underscore the significance of establishing sensing data resources.

Mehrani et al. investigated sampling rates for active biosensors in wireless body area networks [34]. They propose two deep learning models, based on adaptive neuro-fuzzy interference systems and long short-term memory networks, demonstrating their efficacy in reducing communication and energy costs while improving forecast accuracy through simulation experiments.

Arano-Martinez et al. optimized biosensing performance by integrating machine learning models with optical biosensors to estimate different nonlinear optical interactions efficiently [35].

Idrees et al. conducted a detailed study focusing on the utilization of explainable machine learning algorithms to enable biosensors in identifying potential breast cancer diagnosis biomarkers [36]. Their biosensors offer precise predictions and rational explanations for diagnostic outcomes.

### 2.2. Traditional Machine Learning Methods for Cancer Survival Prediction

The decision tree method relies on rules to make predictions, offering high interpretability and effectiveness in handling features with mixed data types. Assegie et al. applied the decision tree approach to a highly imbalanced dataset, revealing satisfactory performance, though inferior to the AdaBoost algorithm [37]. Momenyan et al. compared decision trees with logistic regression on a survival rate dataset, concluding that decision trees provide more accurate predictions [38]. Juneja et al. proposed a weighted decision tree method, enhancing accuracy through feature selection and weighting [39].

Random forest excels with datasets featuring numerous features. Octaviani et al. employed random forests to predict cancer types from the Wisconsin Breast Cancer Database, with impressive results [40]. Nguyen et al. conducted a similar study, achieving remarkable performance after feature selection [10]. Montazeri et al. compared random forests with traditional methods for predicting patient survival, demonstrating superior accuracy [41].

Support vector machines (SVM) are suitable for small, non-linearly separable datasets. Huang et al. utilized multiple SVM classifiers to predict breast cancer, observing improved performance compared to single classifiers [42]. Kim et al. developed a SVM-based prognostic approach for breast cancer recurrence, outperforming other models [43]. Goli et al. employed Support Vector Regression (SVR) to forecast survival time, enhancing accuracy through feature selection [44].

Pan et al. proposed an innovative approach for analyzing multi-view data, termed low-rank tensor regularized graph fuzzy learning. This method incorporates Jensen–Shannon divergence to capture nonlinear structures, presenting promising applications for breast cancer data management [45].

Hassanzadeh et al. proposed a graph-based semi-supervised learning approach utilizing Laplacian support vector machines, achieving more precise cancer survival predictions through the effective utilization of labeled and unlabeled data [46].

### 2.3. Deep Learning Methods for Cancer Survival Prediction

Multilayer Perceptron (MLP) serves as a versatile neural network, capable of functioning as the final component in a deep learning network structure or as a standalone model. Mojarad et al. employed a simple MLP to predict cancer progression based on tumor markers, achieving accurate and reliable performance through k-fold cross-validation testing [47]. Salehi et al. introduced two ensemble MLP learning models, namely Mixture of MLP-expert and MLP Stacked Generalization, both of which outperformed single MLP models according to cross-validation evaluation results [48]. Rezaeipanah et al. combined MLP with multi-stage weight adjustment to enhance diagnosis accuracy, with the resilient backpropagation algorithm demonstrating the best performance among the three algorithms implemented [49].

Breast cancer prediction using medical images is facilitated by Convolutional Neural Networks (CNNs). Ting et al. utilized a CNN to classify patients as malignant, benign, or healthy based on medical images, achieving superior performance without manual segmentation compared to other methods [15]. Chen et al. employed multi-view vision transformers to capture long-range relationships in multiple mammograms, achieving higher classification performance than alternative methods by integrating local and global transformer representations [50]. Li et al. introduced a concatenation autoencoder (ConcatAE) to integrate multi-omics data representations, achieving optimal performance in breast cancer survival prediction by combining DNA methylation and miRNA expression data [51].

Due to the scarcity of large breast cancer image datasets, some studies utilize data augmentation or transfer learning techniques to train CNN models effectively. Lim et al. employed CNN with data augmentation for breast cancer cell classification, achieving high accuracy across various metrics [16]. Nawaz et al. utilized a CNN pre-trained on ImageNet and fine-tuned it for multi-class breast cancer diagnosis, outperforming human performance [52]. Masud et al. compared the performance of eight pre-trained CNN models, finding optimal configurations for each model [53].

Recurrent neural networks (RNNs) are commonly utilized in breast cancer prediction models. Yao et al. utilized a CNN and RNN concurrently to learn image representations, with RNN capturing temporal relationships between image pixels and demonstrating advantages in parallel processing [17]. Srikantamurthy et al. proposed a hybrid CNN-LSTM architecture for breast cancer classification, with CNN learning image representation and LSTM performing further processing, outperforming DNN models lacking a RNN component [18]. Shawni et al. employed stacked recurrent neural networks to analyze patients’ health records chronologically, achieving superior prediction performance compared to alternative methods [54]. Bichindaritz et al. incorporated biLSTM into their model for survival rate prediction using gene expression data, demonstrating significant enhancements through auxiliary task integration and adaptive weight adjustments [55].

### 2.4. Graph Neural Network Methods for Cancer Survival Prediction

Graph neural networks have received limited attention in the domain of breast cancer prediction. This section provides a comprehensive overview of research not only focused on breast cancer prediction but also encompassing other cancer types.

Among the various graph neural networks employed for cancer prediction, GCN stands out as one of the most commonly utilized. Wang et al. employed genomic and clinical data to construct two biosensor similarity networks, retaining only the edges common to both networks. Their approach involved leveraging multiple data types as node features and applying GCN to construct the model. Experimental results indicated that their proposed model achieved higher accuracy and lower standard deviation compared to alternative methods [23].

In another study, Zhang et al. introduced a hybrid method for breast cancer prediction by amalgamating GNN and CNN. They employed CNN to extract high-level representations from abnormal breast images and subsequently built a biosensor similarity graph based on these representations. Utilizing GCN for semi-supervised learning on the graph, they augmented the model with batch normalization, dropout, and rank-based stochastic pooling, thereby surpassing other architectural frameworks in performance metrics [56].

Chereda et al. devised a GNN-based model for breast cancer prediction wherein they mapped gene data to protein-protein interaction (PPI) networks for individual patients, undertaking graph-level learning. To furnish an explanation for predictions, they employed graph layer-wise relevance propagation to delineate a molecular subnetwork for each patient [57].

Gao et al. proposed an innovative approach for predicting survival rates, amalgamating various relationship types to construct graphs. They developed modules capable of learning diverse representations from these relationships, subsequently amalgamating them for predictions [58]. Additionally, they constructed bipartite graphs for patient–gene and patient–CNA relationships, employing GNN to learn patient embeddings separately from both graphs before combining them with clinical data for survival rate predictions, outperforming other methods [59].

Li et al. constructed a heterogeneous supra-graph by amalgamating three types of data and three types of prior knowledge graphs, employing graph attention networks for graph-level learning to classify cancer molecular subtypes. Their performance evaluations on real-world datasets surpassed baseline benchmarks [60].

Ren et al. created patient similarity networks using multi-omics data, utilizing GCN to generate high-level node representations for each network, which were combined using attention mechanisms to predict cancer types [61].

Furtney et al. constructed four distinct graphs from multi-view data, employing GCN to fuse information sources for improved representations, subsequently utilized for predicting breast cancer molecular subtypes [62].

Hao et al. introduced a multi-type data joint learning (MDJL) method for predicting cancer survival, involving the creation of a combined patient similarity matrix from multi-view data followed by GCN application, demonstrating superior effectiveness compared to baseline approaches [63].

Graph attention networks (GATs) offer the capability to assign varying weights to neighbor nodes when aggregating information from them in cancer graph structural data [24]. Qiu et al. proposed a GNN-based method for cancer prediction, involving the construction of a biosensor similarity graph based on feature distances, utilizing a gated graph attention network (GAT) for prediction. Feature selection prior to model training significantly enhanced performance [24]. Wang et al. provided a graph attention network-based approach to analyze an RNA-disease bipartite graph encompassing diseases, including breast cancer. Their model, tested on three datasets, outperformed existing models [26]. Li et al. developed a cell classification model utilizing a k-nearest neighbor algorithm to construct the cell graph based on spatial correlation. Leveraging class-rated and geometric features fed to a graph attention neural network, their proposed method exhibited superior prediction performance and interpretability compared to alternatives [64].

### 2.5. Characteristics Comparison of Different Methods

We have presented a comparison of different methods’ characteristics in Table 1 to showcase the benefits of our proposed approach. Upon examination, we noticed a variety of techniques being employed: some rely on traditional learning algorithms, some delve into deep learning methods, and others make use of graph neural networks. While many methods adopt a multi-view approach, they draw from different omics data sources. Most of these methods employ one-way learning for model training, with only ConcatAE utilizing a two-way learning approach. However, it is worth noting that ConcatAE employs self-supervised learning to generate high-level representations from raw features, without considering gene interactions or patient similarity.

MDJL also incorporates gene expression and patient similarity, but it exclusively utilizes multi-omics data to create diverse patient similarity graphs. Subsequently, it applies GCN on these graphs using semi-supervised learning, without considering gene interactions. In contrast, our method treats gene interaction as one perspective and patient similarity as another, diverging from other methods that treat different omics data as separate entities. The advantage of our method lies in its ability to employ diverse learning approaches to extract more information from both the gene interaction and patient similarity perspectives. This leads to improved prediction performance, particularly when dealing with small datasets.

## 3. Smart Biosensor with Multi-View Multi-Way Graph Learning

In this section, we will introduce our proposed smart biosensor and each of its intelligent components in detail. Firstly, we will give an overview of the smart biosensor architecture and the survival prediction process. After that, we will focus on explaining the function of every intelligent component of the biosensor.

The Learning Center is the most important component, which executes multi-view multi-way graph learning. The main tasks of the Learning Center include feature selection, graph construction, clustering and pseudo label assignment, self-supervised learning on gene interaction graph (GIG), semi-supervised learning on biosensor similarity graph, and model optimization.

### 3.1. Architecture and Prediction Process of Our Proposed Smart Sensor

**Figure 1:**
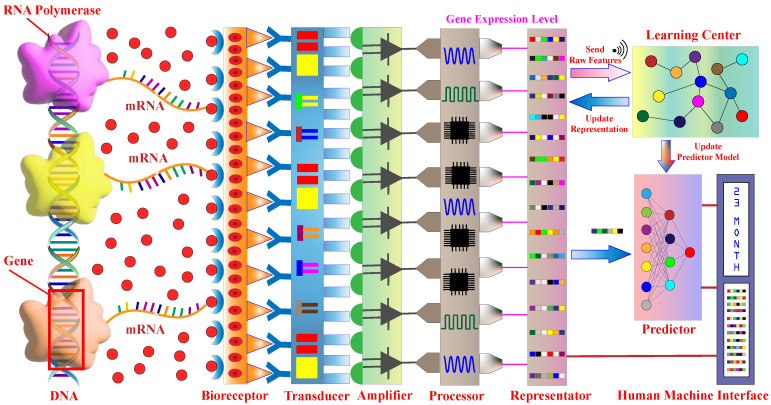
Architecture of our proposed smart biosensor.

Our proposed smart biosensor consists of eight components, namely the Bioreceptor, Transducer, Amplifier, Processor, Representator, Learning Center, Predictor, and Displayer. The architecture of this smart biosensor is shown in Figure 1. Bioreceptor, Transducer, Amplifier, Processor, and Displayer are basic components of traditional biosensors. The functions of these basic components are summarized below.

- *Bioreceptor*. The bioreceptor is utilized to detect biological events, and in our smart biosensor, it is used to detect the production of target mRNAs from genes.
- *Transducer*. The transducer converts biochemical signals from the bioreceptor to measurable physical signals, like electrical or optical signals.
- *Amplifier*. The signals generated by the transducer are typically weak, and amplifiers are used to increase their measurability.
- *Processor*. The amplifier generates signals which are then processed by the processor to extract information related to the sensor task. In our smart sensor, the processor is used to calculate the gene expression level, which is the mRNA production level from genes.
- *Human Machine Interface*. The human machine interface shows readable formats for the processed signal in our smart biosensor, including predicted survival time and gene expression levels.

Our proposed intelligent components—Representator, Learning Center, and Predictor—can naturally utilize various machine learning models. The main function of each component is as follows.

- *Representator*. The representator of our smart biosensor has two primary functions. Firstly, it processes the levels of gene expression generated by the processor component and converts them into a vector which serves as the raw feature of the patient. This vector is then sent to the learning center. Secondly, it receives the learned high-level representation from the learning center and stores it as the final representation of the patient. Once this is done, it feeds the final representation to the predictor to predict the survival time.
- *Learning Center*. The learning center is a critical component of our proposed biosensor. It is a remote computing service that all biosensors share. The learning center executes large neural network models and receives data from multiple sensors to jointly learn high-level representations of biosensors/patients and their prediction models.
- *Predictor*. The predictor uses a compact neural network model to predict the survival time of breast cancer patients. The model’s parameters are learned in the learning center, and every smart biosensor updates the model periodically by downloading the new parameters from the learning center.

**Figure 2:**
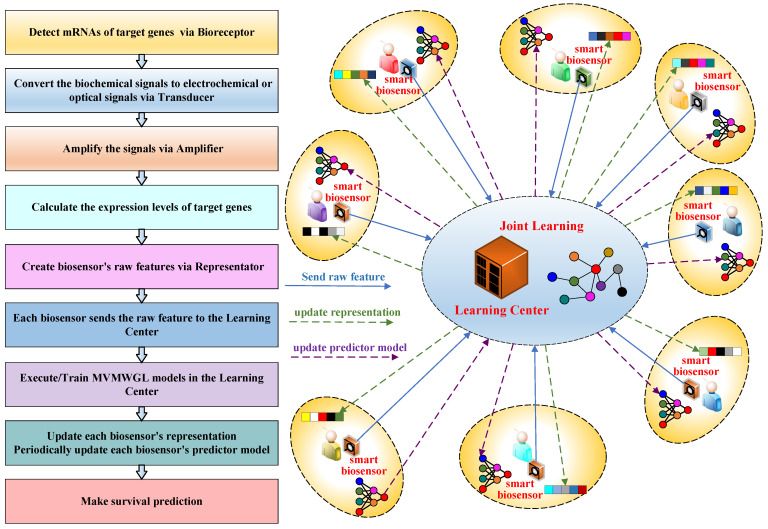
Prediction process of our proposed smart biosensor.

Our proposed biosensor architecture enables us to gather gene expression-level data from multiple patients. These data are then jointly used to learn complex neural network models on the learning center, resulting in accurate predictions. Figure 2 illustrates the prediction process of our smart biosensor, and we explain each step in detail below.

- **Step 1**. The bioreceptor detects mRNA transcription from target genes and generates biochemical signals.
- **Step 2**. The transducer converts biochemical signals into physical signals that can be measured by digital devices.
- **Step 3**. The amplifier boosts transducer signals for better measurement accuracy.
- **Step 4**. The processor calculates the mRNA production levels of target genes.
- **Step 5**. The gene expression levels are organized into a vector form of raw features and sent to the learning center.
- **Step 6**. The learning center is responsible for receiving a large amount of data from various biosensors. It then executes the MVMWGL models to generate new high-level representations for each biosensor. If the models require certain parameters to be adjusted, the learning center will retrain the models and send the prediction model parameters back to each biosensor.
- **Step 7**. The representator stores high-level representation received from the learning center and sends it to the predictor.
- **Step 8**. The predictor uses the input representation sent by the representation to make a survival prediction. If the learning center sends new parameters, the predictor updates its model parameters before making the survival prediction. The output of the predictor is the evaluated survival time.
- **Step 9**. The human machine interface displays the patient’s predicted survival time along with the gene expression levels produced by the processor to explain the prediction.

It is worth noting some important key points for our proposed smart biosensor. Firstly, we assume that each patient has a single smart biosensor. Therefore, each smart biosensor represents one patient. Secondly, all smart biosensors share the same learning center, which executes joint learning by using all the sensor data it receives. Secondly, the predictors of all smart biosensors store the same survival prediction model to make predictions. Lastly, the prediction model is a part of the large model on the learning center, which is jointly trained with the large neural network model.

In the following sections, we will provide a brief introduction to the *Representator* and *Predictor* components, while delving deeper into the *Learning Center* component.

### 3.2. Representator

The “*Representator*” is an essential component added to smart biosensors, which helps to convert the sensor data of the patient into a calculable representation. To execute prediction tasks, smart biosensors typically use machine learning models that require regular data as inputs. However, the output of traditional biosensors’ processors is not well-organized to serve as inputs for learning models. Instead, it is usually used to feed the displayer to show related digital data. For instance, deep learning models require vectors and matrices as inputs, while graph neural models need both vectors or matrices and graph structure data.

In our proposed smart biosensor, the gene expression levels are wrapped by the representator into a long vector to serve as the raw feature of the patient. Specifically, the raw feature of biosensor *i* is denoted as follows.

(1) $$g_{i}=<g_{i1},g_{i2},\ldots ,g_{in}>$$

The raw feature is sent to the displayer to show its values. Additionally, it is sent to the learning center as input to the neural network models. Once the representator receives the high-level representation from the learning center, it saves the new representation in the memory as a vector.

(2) $$z_{i}=<z_{i1},z_{i2},\ldots ,z_{ik}>$$

The learned representation $\vec{z_{i}}$ is sent to the predictor for breast cancer survival prediction. Note that the raw feature is still saved in the memory by the representator.

### 3.3. Multi-View Multi-Way Graph Learning on the Learning Center

**Figure 3:**
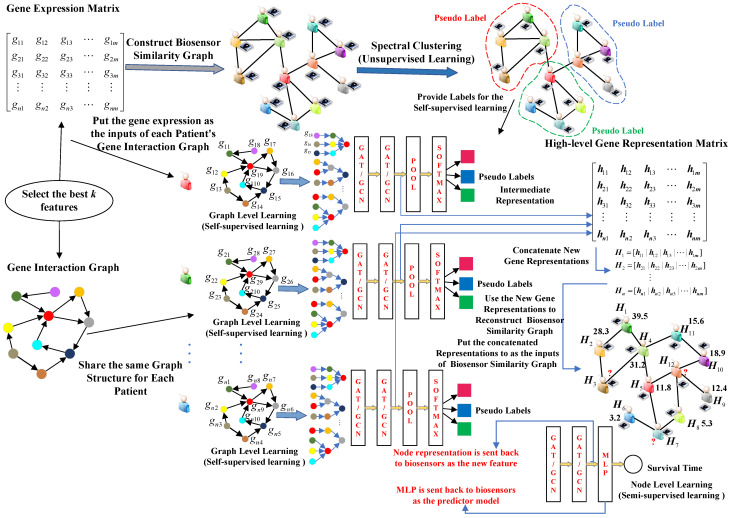
The overall architecture of MVMWGL running on the learning center of smart biosensors.

The learning center gathers a variety of raw features from biosensors measuring gene expression levels. These features are used to create a high-level representation of each patient and a prediction model through multi-view multi-way graph learning. It should be noted that if we have successfully trained the models, we can directly obtain the high-level representation. However, if we have not yet trained the models, we must follow a series of steps to do so. The overall architecture of MVMWGL, which runs on the learning center, is illustrated in Figure 3. The operational steps are as follows.

- *Feature Selection.* In the first step, we select the genes with the highest scores based on calculating each gene’s relevance to survival time.
- *Basic Biosensor Similarity Graph Construction.* Our basic biosensor similarity graph (BBSG) is constructed by computing similarities between patients’ gene expression levels. Biosensors are connected if their similarity exceeds the threshold.
- *Clustering and Pseudo Label Assignment.* We utilize spectral clustering to partition the BBSG into groups and assign a pseudo label to each node based on its group membership.
- *Self-supervised Learning on Gene Interaction Graph.* In this particular step, we create a graph that represents the interaction between genes for each patient. Then, we input the patient’s gene expression level to the graph. Using the gene interaction graph and the patients’ pseudo labels, we leverage a graph convolutional neural network or graph attention neural network to perform self-supervised learning. This helps us to obtain a high-level representation of each patient’s gene expression level.
- *Biosensor Similarity Graph Reconstruction.* We reconstruct the biosensor similarity graph based on the new representation of gene expression level.
- *Semi-supervised Learning on Biosensor Similarity Graph.* We take the learned representation of gene expression levels as inputs for the reconstructed biosensor similarity graph. We label the nodes in the graph with their true survival time and then apply semi-supervised learning to the graph using either graph convolutional neural network or graph neural network. Each smart biosensor’s high-level representation is determined by the node-level output of the layers prior to the final MLP layer, which serves as the prediction model.

In the following sections, we will provide a detailed description of each step. We summarize all the types of graph construction in Section 3.3.2, including basic biosensor similarity graph construction, gene interaction graph construction, and biosensor similarity graph reconstruction.

#### 3.3.1. Feature Selection

To enhance the model’s generalization ability and reduce computation complexity, we use the univariate feature selection approach to select the best genes for training. Each patient’s expression level of gene $g_{j}$ is denoted as $g_{ij}$, and their survival time is denoted as $t_{i}$.

Suppose we have *n* patients and *m* genes. First, we calculate the mean and standard deviation of expression levels for each gene, as well as the mean and standard deviation of survival time for patients.

(3) $$\bar{g_{j}}=\frac{\sum_{i=1}^{n}g_{ij}}{n}$$

(4) $$sd\left(g_{j}\right)=\frac{\sum_{i=1}^{n}\left(g_{j}^{i}-\bar{g_{j}}\right)}{n-2}$$

(5) $$\bar{t}=\frac{\sum_{i=1}^{n}t_{i}}{n}$$

(6) $$sd\left(t\right)=\frac{\sum_{i=1}^{n}\left(t_{i}-\bar{t}\right)}{n-2}$$

Then, the correlation between $g_{j}$ and the survival time *s* is calculated as follows:

(7) $$\rho_{g_{j},s}=\frac{\sum_{i=1}^{n}\left(g_{ij}-\bar{g_{j}}\right)\times \left(t_{i}-\bar{t}\right)}{sd\left(g_{j}\right)\times sd\left(t\right)}$$

We can calculate the F-values of gene $g_{i}$ by using the correlation coefficient:

(8) $$F_{g_{j}}=\frac{{\left(\rho_{g_{j},t}\right)}^{2}}{1-{\left(\rho_{g_{j},t}\right)}^{2}}*\left(n-2\right)$$

By arranging the F-values in descending order, we can select genes with the highest scores.

#### 3.3.2. Graph Construction

After selecting the most relevant features, we get a set of *k* genes. Each patient has a feature vector represented by $g_{i}=\langle g_{i1},g_{i2},\cdots ,g_{ik}\rangle$. Since gene expression levels have different scales, we must standardize the features before proceeding with the training task.

(9) $$g_{{ij}_{std}}=\frac{g_{ij}-\bar{g_{j}}}{sd\left(g_{j}\right)}$$

We use standardized gene expression vectors to calculate biosensor similarity, defining it as the cosine between patient vectors. The similarity between patient *p* and patient *q*, denoted as $s_{pq}$, is calculated using the formula:

(10) $$s_{pq}=\frac{\sum_{j=1}^{k}\left(g_{{pj}_{std}}\times g_{{qj}_{std}}\right)}{\sqrt{\sum_{j=1}^{k}{\left(g_{{pj}_{std}}\right)}^{2}}\sqrt{\sum_{j=1}^{k}{\left(g_{{qj}_{std}}\right)}^{2}}}$$

We can define the adjacency matrix of the basic biosensor similarity graph (BBSG) as the following.

(11) $$A_{pq}=\left\{\begin{array}{c} 1,\;\;if\;\;s_{pq}\geq \eta \\ 0,\;\;\mathrm{otherwise} \end{array}\right.$$

where η represents the threshold value.

**Figure 4:**
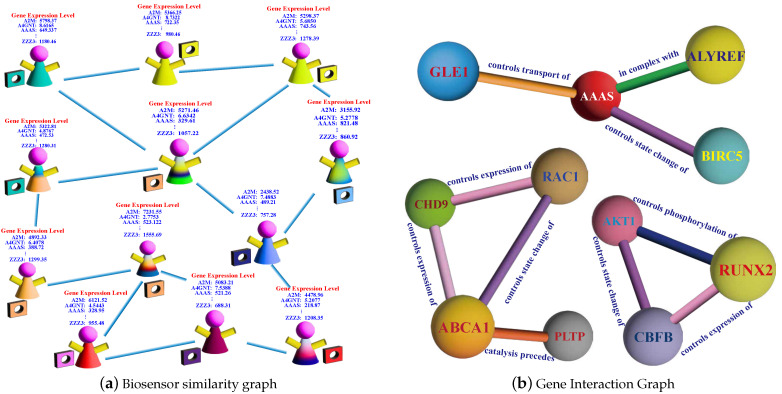
Biosensor similarity graph and gene interaction graph.

In Figure 4a, we depict a biosensor similarity graph, where individual nodes represent patients, and edges signify substantial similarity between pairs of patients. This graph serves as a valuable repository of high-level structural insights not inherently captured by gene expression-level features. As depicted in Figure 3, following the acquisition of high-level gene representations, we proceed to reconstruct the Biosensor Similarity Graph (RBSG) using these updated representations. The construction process for RBSG remains consistent with that of the basic biosensor similarity graph (BBSG).

Figure 4b shows an example of Gene Interaction Graph (GIG), which captures relationships, with each edge representing the influence of a source gene on a target gene’s expression, transport, phosphorylation, etc., or collaborative efforts in complex protein production. The gene interaction graph incorporates complex structural information, which play distinct roles in affecting survival time, and such structural insights are not conveyed by independent gene expression level features.

#### 3.3.3. Clustering and Pseudo Label Assignment

After establishing the foundational biosensor similarity graph, we employ a spectral clustering algorithm to partition patients into distinct groups. The degree matrix of the biosensor similarity graph is denoted as $D_{pq}$, where each element is defined as:

(12) $$D_{pq}=\sum_{q}-A_{pq}$$

Additionally, the Laplacian matrix of the biosensor similarity graph is denoted as $L_{pq}$, and its elements are defined as:

(13) $$L_{pq}=\left\{\begin{array}{c} D_{pq}-A_{pq},\;\;if\;p=q\\ -A_{pq},\;\;\;\;\;\;\;\;\;\;\;\;\;if\;p\neq q \end{array}\right.$$

By conducting eigencomposition on the Laplacian matrix $L_{pq}$, we obtain the following outcome:

(14) $$L_{pq}=Q\Lambda Q^{T}$$

Here, the columns of *Q* represent eigenvectors, and the diagonal elements of Λ correspond to eigenvalues. We select the *k* smallest eigenvalues and their associated eigenvectors, using them in a k-means clustering algorithm to partition patients into distinct groups. Each patient, denoted as *i*, is then assigned a pseudo label $c_{i}$ based on their group membership.

**Figure 5:**
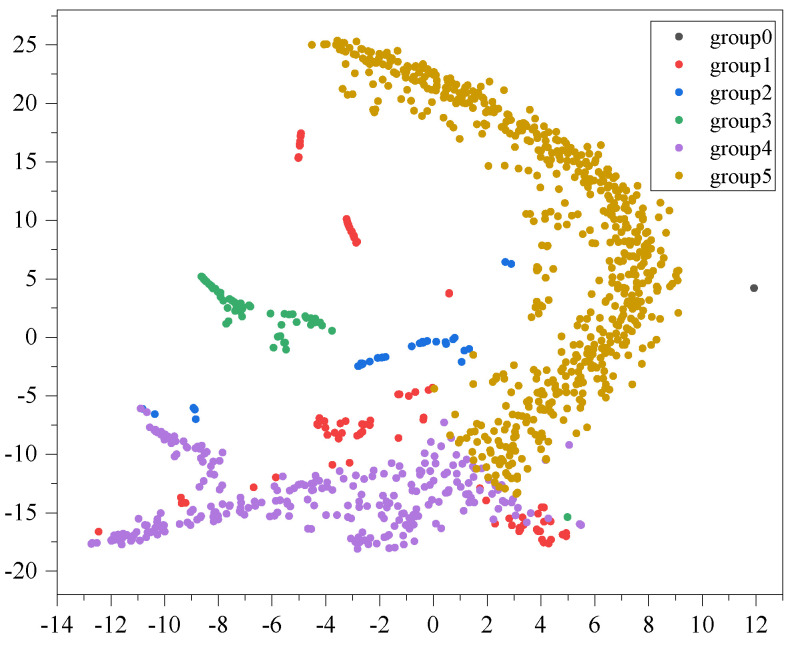
t-SNE visualization of clustering BBSG.

We illustrate the clustering results of BBSG using the t-SNE approach in a two-dimensional space, as depicted in Figure 5. In this example, patients are categorized into five distinct groups, comprising both larger and smaller clusters. The visualization reveals clear structural patterns in the clustering results, with noticeable separation among different groups. This observation indicates the presence of distinct community structures among patients.

#### 3.3.4. Self-Supervised Learning on Gene Interaction Graph

All patients share a common Gene Interaction Graph (GIG) structure, although their gene expression levels vary. By inputting these values into the GIG, we can conduct a graph-level learning task across these patients.

In this study, we leverage GCN and GAT methodologies to acquire high-level representations of genes. The raw input feature for gene *j* in patient *i* is its expression level, denoted as $g_{ij}$, a scalar numerical value. From these initial inputs, we iteratively update gene representations using either GCN or GAT. If we choose GCN, each iteration involves the following operations.

Define the adjacency matrix of the gene interaction graph as *M*. By introducing self-loops, a modified matrix $\hat{M}=M+I$ is obtained. The degree matrix of the gene interaction graph is denoted as D. Normalizing the matrix M using $D^{-1/2}$ and adding an identity matrix results in a new normalized matrix $\hat{M}$, given by ${\hat{D}}^{1/2}\hat{M}{\hat{D}}^{1/2}=D^{1/2}MD^{1/2}+I$, where ${\hat{D}}_{ii}=\sum_{j}M_{ij}$. For each patient, the initial depiction of gene *i* corresponds to its standardized expression level value, specifically denoted as $h_{i}^{0}=g_{i_{std}}$. The representations of genes for each patient in the *l*−th layer constitute a matrix $H^{l}$. At each layer, we update these representations through the application of the following operation.

(15) $$H^{l+1}=\mathrm{ReLU}\left({\hat{D}}^{1/2}\hat{M}{\hat{D}}^{1/2}H^{l}W^{l}\right)$$

The updating process for a specific gene *i* can readily be deduced from the aforementioned formulation:

(16) $$h_{i}^{l+1}=\mathrm{ReLU}\left(\sum_{j\in N(i)\cup \{i\}}\frac{h_{j}^{l}}{\sqrt{d_{i}d_{j}}}W^{l}\right)$$

In the utilization of the graph attention network, the formulation for updating the representation of gene *i* undergoes the following modification:

(17) $$h_{i}^{l+1}=\mathrm{ReLU}\left(\sum_{j\in N(i)\cup \{i\}}\alpha_{i,j}W^{l}h_{j}^{l}\right)$$

where $\alpha_{i,j}$ is called attention coefficients, which is computed as follows:

(18) $$\alpha_{ij}=\frac{\exp\left(\mathrm{LeakyReLU}\left(a^{T}\left[W^{l}h_{i}^{l}\left|\right|W^{l}h_{j}^{l}\right]\right)\right)}{\sum_{k\in N(i)\cup \{i\}}\exp\left(\mathrm{LeakReLU}\left(a^{T}\left[W^{l}h_{i}^{l}\left|\right|W^{l}h_{k}^{l}\right]\right)\right)}$$

where || represents the concatenation operation.

After getting the representations of all genes through *l* layers updating, we merge them together via a global mean pooling operation.

(19) $$h^{l}=\frac{\sum_{i=1}^{k}h_{i}^{l}}{k}$$

Finally, a softmax function is used to predict the pseudo label:

(20) $$\tilde{c}=\mathrm{Softmax}\left(\Theta h^{l}\right)$$

This self-supervised learning task aims to enhance scalar gene expression levels ($g_{i}$) by transforming them into dense vectors ($h_{i}^{l}$). By concatenating the final layer representations of all genes, we create a concise patient feature for survival predictions.

(21) $$H=\left[h_{1}^{l}\left|\right|h_{2}^{l}\left||\cdots |\right|h_{k}^{l}\right]$$

#### 3.3.5. Semi-Supervised Learning on Biosensor Similarity Graph

Leveraging the patient’s new feature, we initiate the reconstruction of the biosensor similarity graph. The reconstruction process parallels the BBSG construction but involves calculating similarity using the standardized vector $h_{std}$ instead of $g_{std}$.

(22) $$s_{pq}=\frac{\sum_{j=1}^{k}\left(h_{j_{std}}^{p}\times h_{j_{std}}^{q}\right)}{\sqrt{\sum_{j=1}^{k}{\left(h_{j_{std}}^{p}\right)}^{2}}\sqrt{\sum_{j=1}^{k}{\left(h_{j_{std}}^{q}\right)}^{2}}}$$

Similar to our approach in self-supervised learning on gene interaction graphs, we apply GCN and GAT for this semi-supervised learning task on the reconstructed biosensor similarity graph. In this context, nodes signify patients, not genes, and we denote the representation of each layer as $z_{i}^{k}$, with the initial representation $z_{i}^{0}=H$. Hence, the representation update process in each iteration for GCN and GAT is defined as follows, respectively:

(23) $$z_{i}^{k+1}=\mathrm{ReLU}\left(\sum_{j\in N(i)\cup \{i\}}\frac{z_{j}^{k}}{\sqrt{d_{i}d_{j}}}W^{k}\right)$$

(24) $$z_{i}^{k+1}=\mathrm{ReLU}\left(\sum_{j\in N(i)\cup \{i\}}\alpha_{i,j}W^{k}z_{j}^{k}\right)$$

We refer to this task as semi-supervised learning because, when a node aggregates information from its neighbors, it includes not only those neighbors in the training set but also those in the test set. Note that the high-level representation $z_{i}^{l}$ of the last layer is sent back to the corresponding biosensor.

To predict the patient’s survival time, the final layer consists of a Multilayer Perceptron (MLP):

(25) $$\tilde{t_{i}}=\mathrm{MLP}\left(z_{i}^{l}\right)$$

**Figure 6:**
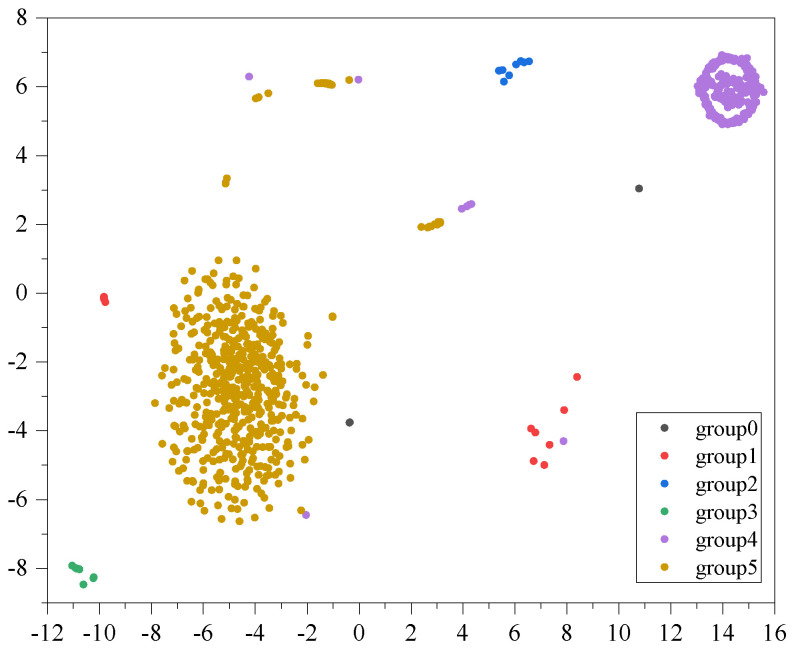
t-SNE visualization of clustering RBSG.

Instead of utilizing the original gene expression level as the patient’s inputs, we employ the gene representation that was acquired through the gene interaction graph as the inputs. The t-SNE visualization result of clustering RBSG is depicted in Figure 6, illustrating more distinct community group structures. The reason for this is that the original features are independent scalar values, and they have less structural information compared to the gene representation that has been learned.

#### 3.3.6. Optimization and Algorithm Design

In order to effectively perform both self-supervised and supervised learning tasks, it is imperative to design appropriate loss functions and optimization algorithms.

For the self-supervised learning task, which entails a classification problem, we employ the CrossEntropy loss. Specifically, we consider a patient *i* and the probability of assigning her to the class *j*, which is denoted as follows:

(26) $${\tilde{c}}_{ij}=\mathrm{Softmax}{\left(\Theta h^{l}\right)}_{ij}$$

For a batch *B* comprising of *b* patients, their loss is computed using the following formula:

(27) $$L_{gig}=-\frac{1}{b}\sum_{i\in B}\sum_{j=1}^{k}c_{ij}\ln\left({\tilde{c}}_{ij}\right)+\lambda_{gig}\phi \left(\Theta \right)$$

For the supervised learning task, which involves regression problems, we make use of mean square error (MSE) loss. In this case, for a batch *B* containing *b* patients, their MSE loss is calculated as follows:

(28) $$L_{bsg}=\frac{1}{b}\sum_{i\in B}{\left(t_{i}-{\tilde{t}}_{i}\right)}^{2}+\lambda_{bsg}\phi \left(\Theta \right)$$

Both $\lambda_{gig}\phi \left(\Theta \right)$ and $\lambda_{bsg}\phi \left(\Theta \right)$ in the two loss functions mentioned above are regularization terms.

We have developed three key algorithms that we would like to highlight in our work. These algorithms are the spectral clustering and pseudo label assignment algorithm (Algorithm 1), the self-supervised learning training algorithm on the GIG (Algorithm 2), and the supervised learning training algorithm on the BSG (Algorithm 3).

Algorithm 1 is an unsupervised learning algorithm that does not require a parameter updating step. We employ the cluster-qr method for patient clustering, which directly extracts clusters from eigenvectors by performing QR factorization. Algorithm 2 uses the pseudo labels, which are the outputs of Algorithm 1, as inputs. In each iteration, we update the parameters using a gradient descent-based method, and the gradient is provided in this algorithm. The outputs of Algorithm 2 are the final layers’ representations, which serve as inputs for Algorithm 3. Algorithm 3 is a node-level task that does not necessitate a pooling layer. We also provide a guide on how to calculate the gradient.
**Algorithm 1:** Spectral clustering and pseudo label assignment
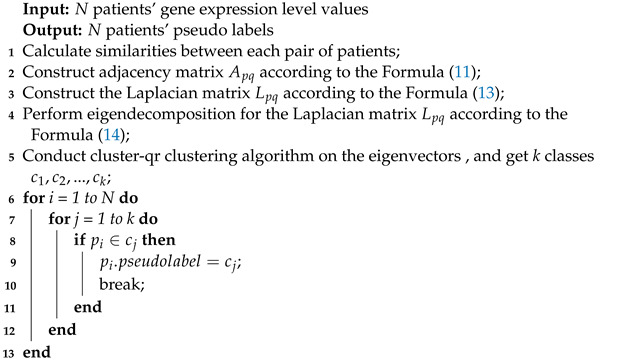


**Algorithm 2:** Self-supervised learning training algorithm on the GIG

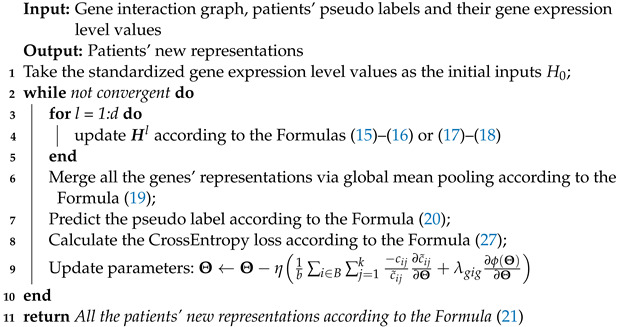



**Algorithm 3:** Supervised learning training algorithm on the BSG

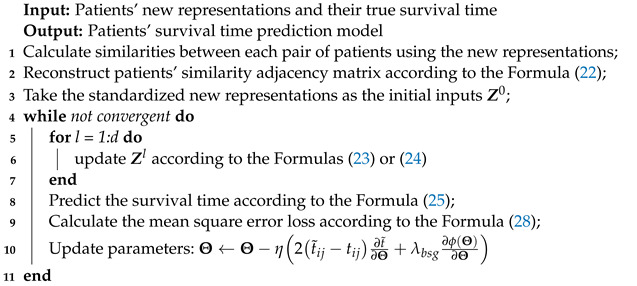



### 3.4. Predictor

The task of semi-supervised learning on the biosensor similarity graph involves each node representing a patient or a biosensor. This is a node-level task that trains a graph neural network model, with the last layer being an MLP. The MLP for the biosensor *i* is denoted as ${\mathrm{MLP}}_{local}^{i}$, while the MLP in the learning center is referred to as ${\mathrm{MLP}}_{remote}$. We design the predicting and model updating process to efficiently make accurate prediction, which is described as follows.

- When it is time for model updating, each predictor requests the learning center for the new prediction model as they all have the same updating cycle.
- If the learning center has recently trained the model, it sends back the MLP layer as the prediction model to the biosensor (${\mathrm{MLP}}_{local}^{i}\leftarrow {\mathrm{MLP}}_{remote}$). Otherwise, it sends a ‘no updating’ signal to the biosensor.
- The representator sends high-level representation to the predictor.
- The predictor receives the high-level representation. If the updating time has not arrived, it directly makes the survival prediction. Otherwise, it first pulls the new model from the learning center and then makes the prediction.

## 4. Experiments

In this section, we will showcase the experimental outcomes of the learning models that can be used in the learning center. Firstly, the datasets, evaluation metrics, and baselines will be introduced. Subsequently, we will present the overall performance comparison results, which will be analyzed in-depth. Additionally, performance comparison results and analysis will be provided on different survival terms. Finally, the convergence analysis and sensitivity analysis of our proposed method will be presented.

### 4.1. Datasets

This study utilized a dataset created by merging two different types of datasets. One of the datasets contained information about patients, including their expression level data and survival records, which were obtained from cBioPortal [65]. The other dataset contained information about gene interactions and came from Pathway Commons [66].

The dataset of patients contains information on gene expression levels as the raw input and survival time as the output, which is divided into three parts, including training, validation set, and test sets. Each set includes 649, 216, and 217 patients, respectively. Each row of the dataset contains 9288 genes and a label representing the survival time measured in months.

The gene interaction dataset contains information on the relationship between pairs of genes, with each row representing a specific relationship. The dataset includes 12,324 genes and 12 different types of relationships. After merging this dataset with another, the resulting dataset includes only 9288 genes and 6 types of relationships. A gene interaction graph can be created by combining data on gene expression levels and interactions. Additionally, we have generated a dataset that includes information on the similarities between patients. Combining this data with gene expression levels creates a biosensor similarity graph.

### 4.2. Evaluation Metrics

We evaluate our method and baselines using two popular regression model evaluation metrics: mean square error (MSE) and mean absolute error (MAE). MSE calculates the average squared differences between predicted and actual values while MAE calculates the average absolute differences between predicted and actual values.

(29) $$\mathrm{MSE}=\frac{\sum_{i=1}^{N}{\left(t_{i}-{\tilde{t}}_{i}\right)}^{2}}{N}$$

(30) $$\mathrm{MAE}=\frac{\sum_{i=1}^{N}\left|t_{i}-{\tilde{t}}_{i}\right|}{N}$$

### 4.3. Baselines

We compared MVMWGL running on the learning center with 11 non-GNN-based methods and 4 GNN-based methods, as described below. For clarity, we assign symbols to each method for ease of plotting method names in figures. For example, “T1” refers to Linear Regression and “T10” refers to Decision Tree.

- T1—**Linear Regression** [9]: it is a basic model that uses the weighted sum of features for predictions.
- T2—**Ridge Regression** [67]: it is a simple model that incorporates $\ell_{2}$-norm regularization into linear regression.
- T3—**Lasso** [68]: this model is a linear regression that includes $\ell_{1}$-norm regularization.
- T4—**Elastic Net** [69]: it is a linear regression model that incorporates both $\ell_{1}$-norm and $\ell_{2}$-norm regularization.
- T5—**LARS lasso** [70]: it is a lasso model, which obtains the solution through the least-angle regression algorithm.
- T6—**Bayesian Ridge** [71]: assuming a spherical Gaussian prior distribution of parameters, this probabilistic model considers the output to be Gaussian distributed around the weighted sum of features.
- T7—**Tweedie Regressor** [72]: it is a generalized linear model that assumes the condition probability obeys the Tweedie distribution.
- T8—**Kernel Ridge** [73]: this model combines ridge regression with kernel tricks, such as polynomial, RBF, and Laplacian kernels.
- T9—**Gaussian Process** [74]: It is a non-parametric probabilistic model that assumes the function follows a Gaussian distribution.
- T10—**Decision Tree** [37]: using tree structures, the non-parameter model divides the feature space into parts, with each part having a specific output.
- T11—**Multilayer Perceptron** [49]: the model is a fully connected neural network with several hidden layers.
- G1—**GIG-GCN** [23]: This approach utilizes graph convolutional layers on the gene interaction graph to forecast survival time.
- G2—**GIG-GAT** [24]: this method employs graph attention layers on the gene interaction graph to predict survival time.
- P1—**BSG-GCN** [23]: this method uses graph convolutional layers in the model on the biosensor similarity graph for predicting survival time.
- P2—**BSG-GAT** [24]: this model predicts survival time by utilizing graph attention layers on the biosensor similarity graph.

In our proposed method, there are four different configurations depending on the type of graph neural layer used.

- M1—**MVMWGL-GCNCN**: this approach uses graph convolutional layers for both self-supervised learning on the gene interaction graph and supervised learning on the biosensor similarity graph.
- M2—**MVMWGL-GATCN**: this method leverages graph attention and convolutional layers for self-supervised and supervised learning on the gene interaction and biosensor similarity graphs, respectively.
- M3—**MVMWGL-GCNAT**: this approach employs graph convolutional layers for self-supervised learning on the gene interaction graph and graph attention layers for supervised learning on the biosensor similarity graph.
- M4—**MVMWGL-GATAT**: this method utilizes graph attention layers for both self-supervised training on the gene interaction graph and supervised learning on the biosensor similarity graph.

While training these four different models, we set the number of pseudo-labels to 6 and the number of GCN or GAT layers to 2. We selected 2000 genes with the best scores and used their expression levels as the raw features. The number of training epochs for self-supervised learning was set to 40, and the learned representation size for self-supervised learning was set to 4. The number of training epochs for semi-supervised learning was determined by achieving the best performance on the validation set. We used Adam as the optimizer, and the learning rates for both self-supervised and semi-supervised learning models were set to 0.001.

### 4.4. Overall Performance Comparison and Analysis

This section presents the results of our performance comparison on patients across all survival time periods. Table 2 displays the mean square error (MSE) and mean absolute error of all compared methods, including their standard deviation. Kindly take note that the survival time is measured in months.

Upon reviewing the data in Table 2, it is evident that GNN-based methods produced lower MSE values than non-GNN-based methods, and lower MAE values than most non-GNN-based methods. The only exception is Kernel Ridge, which had a lower MAE than GIG-GAT. This suggests that GNN-based methods that utilize the relationships between features or instances can help improve performance. The results indicate that GIG-GCN and GIG-GAT demonstrated a lower mean squared error (MSE) and standard deviation than BSG-GCN and BSG-GAT. However, it is important to note that GIG-GCN and GIG-GAT had a higher mean absolute error (MAE) than BSG-GCN and BSG-GAT.

Our proposed MVMWGL methods, with different configurations, outperform all other methods both in terms of the MSE and MAE metrics. They reduce the MSE by approximately 30% compared to the lowest MSE obtained by other methods. Similarly, the MAE is reduced by about 20% compared to the lowest MAE obtained by other methods. These results suggest that by incorporating both the relationships between features and the relationships between instances, our MVMWGL methods are able to further improve prediction performance.

**Figure 7:**
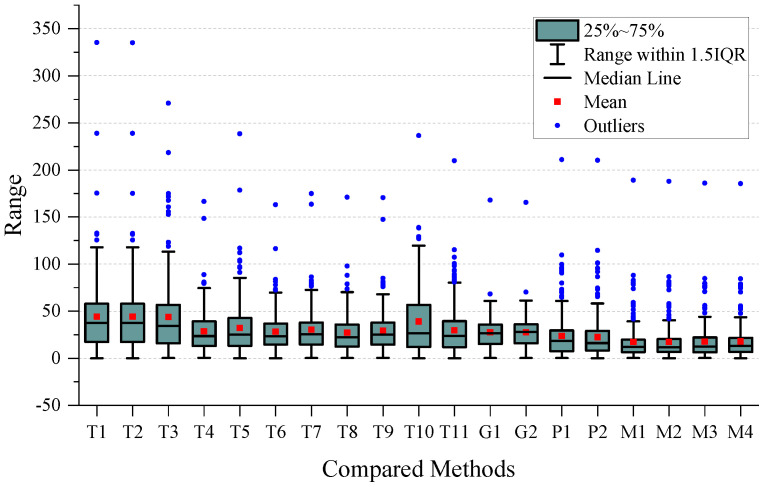
Boxplot of the overall mean absolute error for all the compared methods.

To perform a more detailed analysis of the performance, we created a boxplot of the MAE for all the methods used, which is presented in Figure 7. After examining the plot, we observed that the four types of MVMWGL methods had lower mean and narrower interquartile ranges than other methods. This indicates that MVMWGL not only produces a smaller prediction error, but also provides a consistent prediction error spread within the middle 50% of the data. The results of Table 2 indicate that GIG-GCN and GIG-GAT have the lowest standard deviation. The reason for this is shown in Figure 7, where we can see that they have a small number of outliers, and these outliers are not very far from the mean. BSG-GCN and BSG-GAT measure biosensor/patient similarity based on original gene expression levels, disregarding gene relationships, leading to more unstable prediction errors compared to GIG-GCN and GIG-GAT. The MVMWGL method combines the benefits of GIG-GCN/GAT and BBSG-GCG/GAT, resulting in a low mean and small standard deviation. 

### 4.5. Performance Comparison and Analysis on Different Survival Time Periods

While our proposed method has demonstrated superior performance compared to other baselines across all the survival time periods, it is prudent to conduct a detailed analysis of its performance across different time periods. This will enable us to gain a deeper understanding of the properties of MVMWGL and to make informed decisions about its effectiveness in addressing our research objectives. In this section, we categorize the survival time periods into three terms: short-term (less than or equal to 12 months), medium-term (more than 12 months and equal to 60 months), and long-term (more than 60 months).

Table 3 shows the comparison results of MSE and MAE performance, while Table 4 displays the comparison results of MAE standard deviation. We can see that GNN-based methods have lower MSE and MAE than non-GNN-based methods on the short-term and medium-term data, but not necessarily on the long-term data.

On the short-term data, BSG-GCN and BSG-GAT have the lowest Mean Squared Error (MSE) and Mean Absolute Error (MAE) when compared to other methods, except for MVMWGL-GCNCN which has a slightly lower MSE than BSG-GCN. In addition to BSG-GCN and BSG-GAT, four types of MWMGL methods have the lowest MSE and MAE. GIG-GCN and GIG-GAT do not seem to perform better than MLP on the short-term data. However, MWMGL methods have the lowest standard deviation for both MSE and MAE compared to other methods. Although the MAE standard deviation of GIG-GCN/GAT is as small as that of MVWMGL methods, they have a higher MSE standard deviation than MVWMGL methods. On the short-term data, the MSE standard deviation of MVEMGL is about 45% of the lowest standard deviation among other methods, and the MAE standard deviation is about 30% of the lowest standard deviation among other methods, except for the GIG-GCN/GAT method.

On the medium-term data, MVMWGL methods achieve the lowest MSE and MAE compared to all other methods, along with the lowest standard deviation. The MVMWGL methods reduce the Mean Squared Error (MSE) by at least 67% compared to the lowest MSE achieved by other methods. They also reduce the Mean Absolute Error (MAE) by at least 41% compared to the lowest MAE obtained by other methods. Additionally, their MSE and MAE standard deviations are nearly 60% and 55%, respectively, when compared to the lowest standard deviation obtained among other methods.

**Figure 8:**
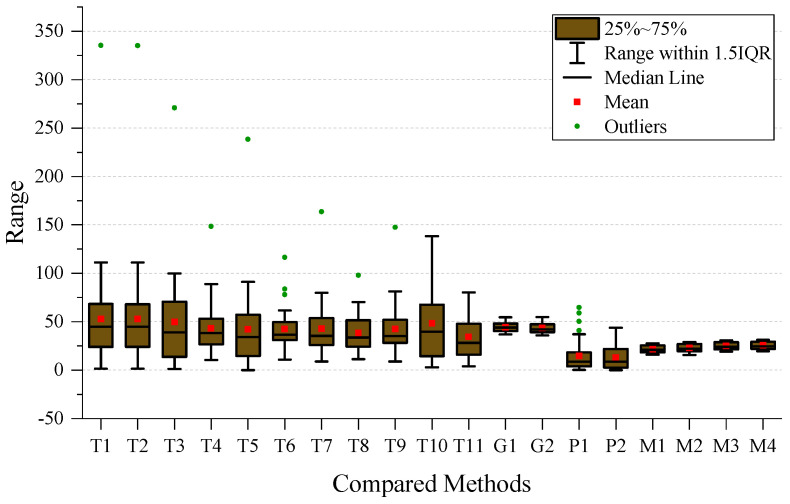
Boxplot of the mean absolute error on short-term data.

**Figure 9:**
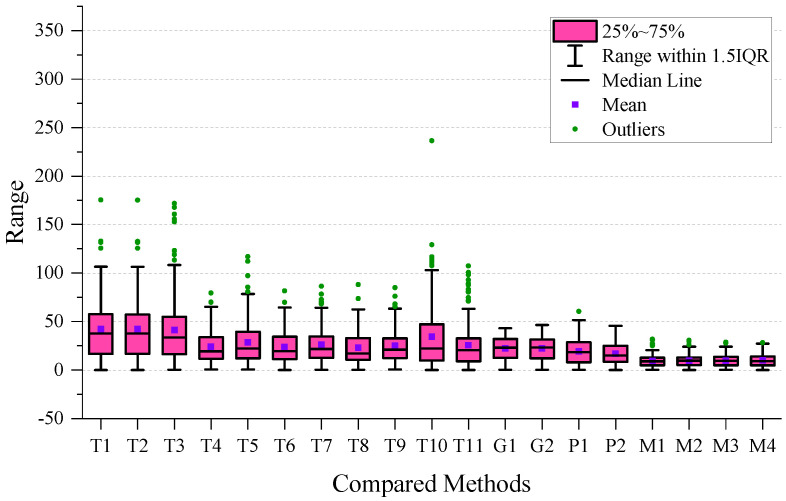
Boxplot of the mean absolute error on medium-term data.

**Figure 10:**
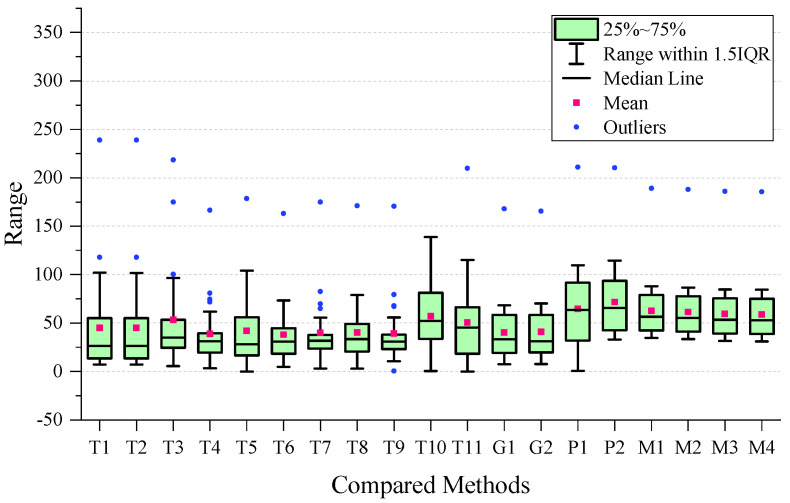
Boxplot of the mean absolute error on long-term data.

To ensure a comprehensive analysis of prediction performance, the Mean Absolute Error (MAE) boxplot has been presented for each time period. The boxplot for short-term results can be found in Figure 8, whereas the boxplot for medium-term results is located in Figure 9. Additionally, the boxplot for long-term results can be found in Figure 10.

On the short-term data, we can observe that the GIG-GCN/GAT and MVMWGL methods have significantly narrower interquartile ranges compared to others, and also have no outliers. However, the mean of GIG-GCN/GAT is higher than that of MVMWGL. BSG-GCN/GAT has a lower mean value than MVWMGL, but its IQR is broader than that of MVMWGL, and it even has some outliers. Among all the compared methods, MVMWGL has the lowest mean value and narrowest IQR on medium-term data, indicating a consistently steady prediction error spread. However, on the long-term data, MVMWGL methods as well as GIG-GCN/GAT and BSG-GCN/GAT display broad IQR and high mean values, which indicate worse performance even when compared to some non-GNN-based methods. 

**Figure 11:**
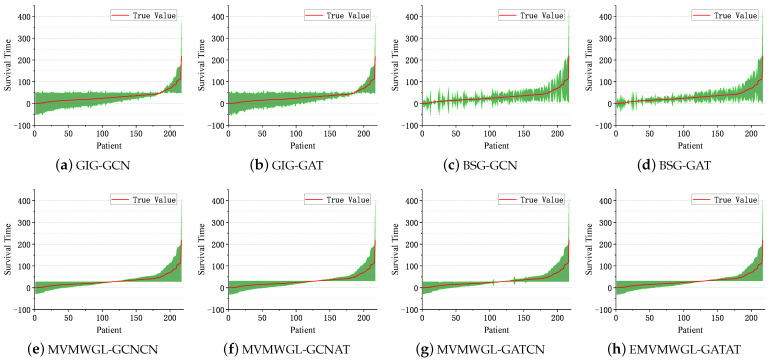
Survival time prediction error bar of all GNN-based methods.

In order to gain a better understanding of the prediction performance of GNN-based methods, we arranged the patients in the test set in ascending order according to their actual survival time. We then created an error bar along this ordered sequence to provide a more intuitive representation of the results. These results are presented in Figure 11. We can observe that MVMWGL methods exhibit narrow error bars for short-term and medium-term while displaying broader error bars for long-term. While GIG-GCN/GAT and BSG-GCN/GAT have broader error bars than MVMWGL over time, there are some differences between them. We can see that the error bars of BSG-GCN/GAT fluctuate more than GIG-GCN/GAT, which means that they have a lower error on average.

Although GNN-based methods are not better than non-GNN-based methods at predicting survival time in the long-term, this does not diminish the importance of adopting them for prediction. This is because the number of patients who have a long-term survival time (more than five years) is quite small, and even if the prediction error is large, leading to urgent treatment, it poses a low risk for these patients.

After analyzing the results of the experiment above, it is found that MVMWGL can significantly enhance the prediction of short-term and medium-term survival time. This indicates that gene interaction plays a crucial role in determining a patient’s survival time. Patients with similar gene interaction tend to have similar survival time, and leveraging their structural information can improve the prediction performance.

### 4.6. Convergence Analysis

In this section, we analyze the convergence property of MVMWGL as well as other GNN-based methods. The data is divided into three parts: the training set, validation set, and test set. We optimize the trainable parameters on the training set, tune the hyperparameters on the validation set and evaluate the prediction performance on the test set. The training epoch is a crucial hyperparameter, and we must decide on the iterative value for both self-supervised learning on the GIG and supervised learning on the BSG. To avoid overfitting in supervised learning, we select the epoch value at which the validation loss is at its minimum. In the case of the MVMWGL method, the training process involves two stages. For the self-supervised learning stage, we choose the epoch value when the validation loss starts to stabilize.

**Figure 12:**
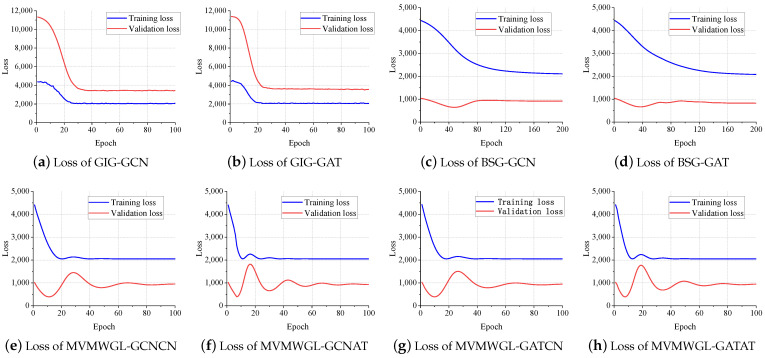
Training and validation loss.

Figure 12 displays the training and validation loss of all the GNN-based approaches. For MVMWGL, only the supervised learning loss on the biosnsor similarity graph is shown.

The training loss and validation loss for GIG-GCN/GAT are very close, while those for BSG-GCM/GAT are comparatively larger. This suggests that GIG-GCN/GAT has a relatively strong bias and weak variance, whereas BSG-GCN/GAT has a weak bias and strong variance. The training loss and validation loss of MVMWGL are a little farther apart than those of GIG-GCN/GAT, but closer than those of BSG-GCN/GAT. This also explains why MVMWGL has a smaller standard deviation than BSG-GCN/GAT, but a higher standard deviation than GIG-GCN/GAT in the previous experimental results.

It is worth noting that GIG-GCN/GAT’s training loss is smaller than its validation loss. However, BSG-GCN/GAT or MVWMGL’s training loss is higher than its corresponding validation loss. Both GIG-GCN and GIG-GAT have very smooth training and validation loss curves with no fluctuations once the loss reaches its lowest point. BSG-GCN and BSG-GAT have smooth and consistent training loss curves, but their validation loss curves display some fluctuations. Our proposed method has a training loss curve with small fluctuations, but the validation loss curve shows apparent fluctuations. This indicates that BSG-GCN/GAT and MVWMGL have better generalization abilities than GIG-GCN/GAT, which explains why BSG-GCN/GAT and MVWMGL perform better than GIG-GCN/GAT.

Our proposed method, MVMWGL, combines the strengths of GIG-GCN/GAT and BSG-GCN/GAT, resulting in better generalization and reduced variation, which leads to the improved prediction performance of survival time.

### 4.7. Sensitivity Analysis

In this section, we conduct a sensitivity analysis of our proposed method to examine the factors that influence the prediction of survival time. We consider three types of factors: the number of pseudo labels, the number of self-supervised learning epochs on the GIG, and the similarity threshold for constructing the BSG.

**Figure 13:**
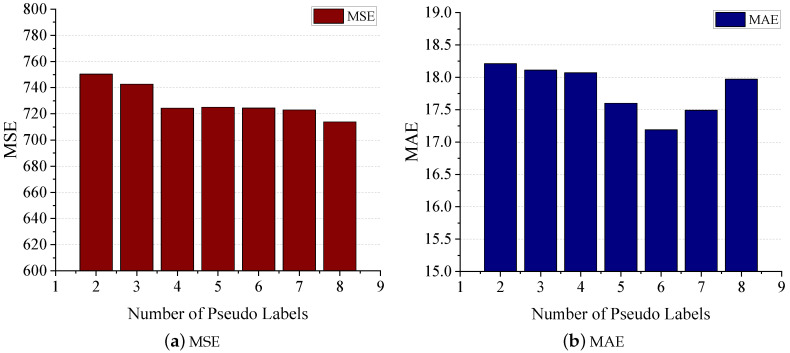
Impact of number of pseudo labels.

The impact of the number of pseudo labels on prediction performance is illustrated in Figure 13. It is noticeable that a small number of pseudo labels results in higher MSE and MAE values. With an increase in the number of pseudo labels, the MSE stabilizes. However, the MAE initially decreases to its lowest point before rising again. This suggests an optimal, moderate number of pseudo labels.

**Figure 14:**
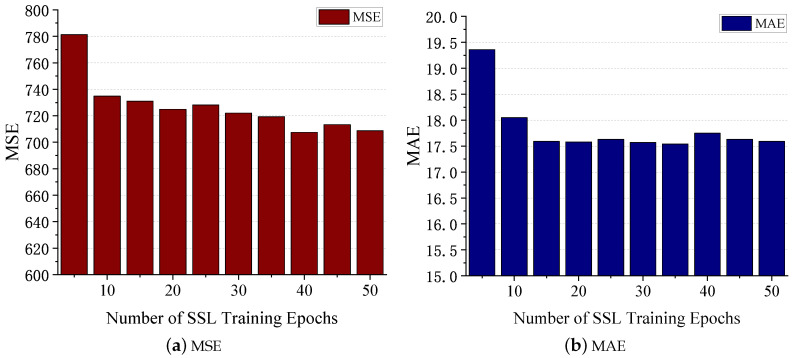
Impact of number of SSL training epochs.

In Section 4.6, we previously discussed that prior to conducting supervised learning on BSG, we iteratively trained the self-supervised learning (SSL) model until the validation loss stabilized. Here, we delve deeper into how the number of SSL training epochs impacts the subsequent supervised learning performance. The results are depicted in Figure 14. As the number of epochs increases, we observe a gradual stabilization of MSE and MAE on the test set, similar to the trend observed in the validation set’s loss. This finding supports the viewpoint that choosing the number of epochs when the validation loss becomes stable is a valid approach.

**Figure 15:**
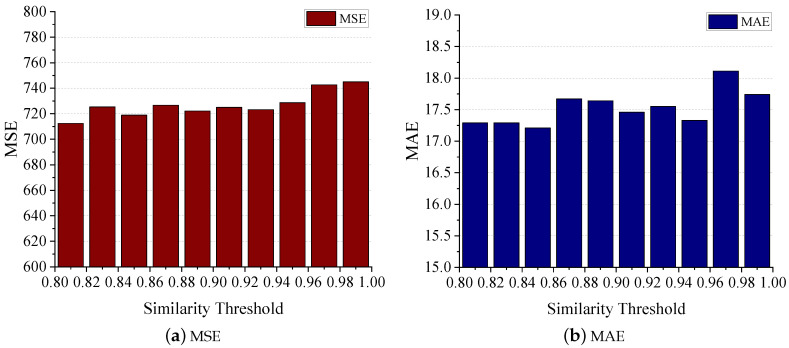
Impact of similarity threshold of BSG.

When constructing the biosensor similarity graph, our process commences with computing the similarity between patients. We then establish connections between them if their similarity exceeds a certain threshold. Consequently, the density of the graph hinges on this similarity threshold, which subsequently affects the efficacy of learning within the graph neural layers. Figure 15 demonstrates how the variation in the similarity threshold impacts the MSE and MAE on the test set. The results indicate that as the threshold increases, both MSE and MAE gradually escalate. This phenomenon could be attributed to the higher threshold causing the graph to become too sparse, thereby hindering nodes from aggregating sufficient information from their neighbors. It is worth noting that the threshold is set to a minimum of 0.8. This precaution is taken because setting the threshold too low could lead to the graph becoming overly dense, resulting in nodes learning similar representations, which hold no significance for the model.

## 5. Conclusions

Biosensors have emerged as valuable tools for cancer detection and predicting survival rates through the integration of machine learning models. In this study, we introduce a novel intelligent biosensor comprising three components absent in traditional designs. The “representator” generates raw features from sensor data and extracts high-level representations from the learning center, while the “predictor” houses a compact neural model downloaded from the learning center to predict survival rates.

The learning center employs complex neural network models to derive high-level representations for biosensors (patients). Accurate prediction of survival time is crucial for tailoring treatment plans in breast cancer, where patients with short-term, medium-term, and long-term prognoses require personalized approaches. Despite the critical role of gene expression levels in many models, existing methods often overlook the interplay between genes and patients. In our proposed smart biosensor’s learning center, we introduce a novel approach termed multi-view multi-way graph neural network learning (MVMWGL). “Multi-view” reflects our consideration of survival time from two perspectives: gene interaction and biosensor similarity, while “multi-way” incorporates three learning paradigms: unsupervised, self-supervised, and supervised learning.

On the learning center of our proposed biosensor, MVMWGL initially constructs the biosensor similarity graph via unsupervised learning to obtain pseudo labels for patients. Subsequently, it employs these labels alongside gene expression levels for self-supervised learning on the gene interaction graph, yielding new gene representations for the subsequent supervised learning task on the biosensor similarity graph. MVMWGL leverages gene interaction, biosensor similarity, and diverse learning paradigms. Our experimental results demonstrate the superiority of our method in survival time prediction, particularly for short-term and medium-term patients.

However, our proposed method has limitations. Firstly, learning models are trained centrally, and each biosensor must periodically receive updated representations and MLP prediction models from the learning center. In cases of numerous biosensors or network delays, inconsistencies may arise. Secondly, our smart biosensor assumes uniform gene interaction graph structures among patients, overlooking individual biological pathways’ influence. Lastly, frequent connections and disconnections between biosensors and the learning center hinder the maintenance of a stable patient similarity graph. Future work will explore breast cancer survival prediction using biosensors with dynamic multi-omics interaction graphs to address these challenges.

## Figures and Tables

**Table 1:** Characteristics comparison of different methods.

Method	Feature Type	View Type	Model Type	Lerning Way
LSVM-GSSL [46]	Gene Expression	Multi-View	LSVM	Semi-Supervised
CNNI-BCC [15]	Images	Single-view	CNN	Supervised
ConcatAE [51]	Gene Expression	Multi-view	AutoEndoer, DNN	Self-Supervised, Supervised
ML_ordCOX [55]	Gene Expression	Multi-view	LSTM, CNN	Supervised
GCGCN [23]	Gene Expression	Multi-view	GCN	Supervised
GGAT [24]	Gene Expression	Single-view	GAT	Supervised
MDJL [63]	Gene Expression, Patient Similarity	Multi-view	GCN	Semi-supervised
MVMWGL	Gene Expression, Patient Similarity	Multi-view	GCN, GAT, Spectral clustering	Unsupervised, Self-supervised, Semi-supervised

**Table 2:** Overall performance comparison.

Method	MSE	MAE
Linear Regression (T1)	3468.64 (±9138.07)	44.1 (±39.04)
Ridge Regression (T2)	3462.90 (±9125.50)	44.06 (±39.01)
Lasso (T3)	3513.67 (±7580.32)	43.79 (±39.95)
Elastic Net (T4)	1339.99 (±2679.24)	28.74 (±22.62)
Lars Lasso (T5)	1912.94 (±4838.24)	32.01 (±29.80)
Bayesian Ridge (T6)	1213.09 (±2288.21)	28.12 (±20.56)
Tweedie Regressor (T7)	1455.69 (±3002.95)	30.22 (±23.29)
Kernel Ridge (T8)	1161.19 (±2353.32)	27.16 (±20.58)
Gaussian Process (T9)	1361.04 (±2714.06)	29.39 (±22.30)
Decision Tree (T10)	2746.06 (±5373.83)	39.53 (±36.57)
Multilayer Perceptron (T11)	1622.66 (±3670.38)	29.67 (±27.25)
GIG-GCN (G1)	1024.79 (±2027.26)	27.15 (±16.95)
GIG-GAT (G2)	1043.29 (±1984.53)	27.54 (±16.88)
BSG-GCN (P1)	1149.02 (±3454.51)	23.50 (±24.43)
BSG-GAT (P2)	1077.36 (±3475.68)	22.28 (±24.10)
MVMWGL-GCNCN (M1)	712.16 (±2696.80)	17.24 (±20.37)
MVMWGL-GATCN (M2)	704.18 (±2646.44)	17.42 (±20.02)
MVMWGL-GCNAT (M3)	695.60 (±2573.58)	17.65 (±19.61)
MVMWGL-GATAT (M4)	694.95 (±2556.80)	17.73 (±19.50)

**Table 3:** We show the performance comparison on different survival time periods.

Method	Short		Medium		Long	
	MSE	MAE	MSE	MAE	MSE	MAE
Linear Regression (T1)	5974.24	52.94	2776.97	42.09	4501.39	44.90
Ridge Regression (T2)	5966.96	52.91	2771.43	42.04	4496.52	44.89
Lasso (T3)	4908.50	49.56	2928.88	41.10	5411.91	53.46
Elastic Net (T4)	2516.26	43.07	895.35	24.14	2619.33	38.93
Lars Lasso (T5)	3536.02	42.12	1332.90	28.37	3456.27	41.82
Bayesian Ridge (T6)	2200.66	42.43	816.41	23.57	2442.07	37.97
Tweedie Regressor (T7)	2607.40	42.92	1021.15	26.04	2702.91	39.88
Kernel Ridge (T8)	1820.95	38.35	781.56	22.81	2741.59	40.09
Gaussian Process (T9)	2435.24	42.57	942.38	25.07	2612.86	39.31
Decision Tree (T10)	3935.11	48.46	2418.05	34.37	4309.07	56.98
Multilayer Perceptron (T11)	1662.81	34.54	1172.96	25.47	4545.02	50.54
GIG-GCN (G1)	1992.14	44.36	602.53	22.04	2651.30	40.30
GIG-GAT (G2)	1930.68	43.58	608.24	22.11	2668.33	40.81
BSG-GCN (P1)	497.23	14.78	541.41	19.14	6097.88	64.77
BSG-GAT (P2)	339.40	12.88	404.47	16.82	6580.72	71.79
MVMWGL-GCNCN (M1)	490.27	21.79	127.14	9.42	4902.21	62.62
MVMWGL-GATCN (M2)	544.86	22.99	128.75	9.60	4742.17	61.32
MVMWGL-GCNAT (M3)	641.39	25.02	131.61	9.76	4508.77	59.39
MVMWGL-GATAT (M4)	665.18	25.49	133.99	9.86	4453.48	58.92

**Table 4:** MAE standard deviation comparison on different survival time periods.

Method	Short		Medium		Long	
	SD-SE	SD-AE	SD-SE	SD-AE	SD-SE	SD-AE
Linear Regression (T1)	18,756.94	56.3	4109.10	31.71	11,472.20	49.86
Ridge Regression (T2)	18,733.07	56.28	4100.07	31.68	11,458.86	49.82
Lasso (T3)	12,331.09	49.52	5180.00	35.21	10,850.96	50.53
Elastic Net (T4)	3803.55	25.72	1181.11	17.68	5528.07	33.22
Lars Lasso (T5)	9503.09	41.98	2123.88	22.98	6744.47	41.32
Bayesian Ridge (T6)	2455.85	20.01	1043.43	16.16	5258.28	31.63
Tweedie Regressor (T7)	4498.95	27.66	1339.45	18.52	6024.06	33.35
Kernel Ridge (T8)	1879.16	18.71	1103.07	16.16	5739.44	33.68
Gaussian Process (T9)	3695.98	24.96	1223.76	17.72	5741.55	32.67
Decision Tree (T10)	5345.14	39.84	5437.09	35.16	4444.31	32.59
Multilayer Perceptron (T11)	1845.47	21.68	2113.45	22.89	8803.85	44.61
GIG-GCN (G1)	445.60	4.93	470.90	10.80	5500.99	32.06
GIG-GAT (G2)	505.50	5.63	481.13	10.93	5357.58	31.67
BSG-GCN (P1)	995.99	16.70	639.73	13.23	8731.78	43.62
BSG-GAT (P2)	565.43	13.17	464.48	11.03	8560.75	37.78
MVMWGL-GCNCN (M1)	172.81	3.93	171.46	6.20	6754.76	31.32
MVMWGL-GATCN (M2)	187.47	4.07	157.98	6.05	6676.42	31.33
MVMWGL-GCNAT (M3)	197.73	3.92	146.07	6.03	6560.27	31.33
MVMWGL-GATAT (M4)	202.06	3.93	145.04	6.07	6534.34	31.33

## Data Availability

Data are contained within the article.
